# Stealth-vaping: a new era of illicit substance misuse? a systematic review and meta-analysis of the prevalence of electronic nicotine delivery systems for the consumption of illicit substances

**DOI:** 10.1192/bjo.2021.686

**Published:** 2021-06-18

**Authors:** Tim Hicks, Rebecca Arrowsmith, Susannah Keill, Eleni Papadakou

**Affiliations:** 1 Dorset Healthcare University NHS Foundation Trust; 2 University Hospital Dorset NHS Foundation Trust; 3 University Hospital Southampton NHS Foundation Trust

## Abstract

**Aims:**

To estimate the prevalence of using Electronic Nicotine Delivery Systems (ENDS) for the consumption of illicit Substances (illegal under UK Law). We hypothesised that this is an increasingly common mode of delivery.

**Background:**

Using ENDS to consume nicotine is increasing in popularity worldwide with a prevalence in the UK of 6% and in the USA 4%-6%. Existing studies have reported that people are switching to vaping because it is felt to be safer than smoking.

However there is also emerging evidence that this mode of consumption is increasingly being used as it is discreet and much less easy to detect, hence sometimes referred to as stealth-vaping. This appears to be driving a switch to vaping to administer substances other than nicotine, notably, but not exclusively cannabis, including concentrated forms of Tetrahydrocannabinol (THC) and synthetic cannabinoids. Anecdotally this practice is known to be occurring in psychiatric inpatient settings.

This is against a backdrop of the uncertain long-term effects of vaping and the emergence of case reports of the death of otherwise healthy young persons after using ENDS to consume cannabis.

**Method:**

Search strategy: MEDLINE , EMBASE, Cochrane Database of Systematic Reviews, Grey Literature using Medical Subject Headings (MeSH), text words relating to vaping of drugs and hand searching journals.

Statistical methods: Synthesis of data was performed using inverse variance with double arcsine transformation in MetaXL. Heterogeneity was assessed with the Cochran's Q and I^2^.

**Result:**

From 970 abstracts, 61 papers were selected for full text review, 18 met the inclusion criteria. The total study population for the outcome of ENDS nicotine users who also use ENDS for the consumption of illicit substances was 9098. There was significant heterogeneity with a random effects model prevalence of 17% (95%CI 7%-32%). The total study population for the outcome of cannabis users who use ENDS to consume cannabis was 52708. There was significant heterogeneity with a random effects model prevalence of 23% (95%CI 12%-37%).

**Conclusion:**

The use of ENDS to consume illicit substances is concerning as it appears to be relatively common practice. This was most notable in studies of existing cannabis users, younger people and medical marijuana users.

Given the uncertainty of long term health consequences and poor understanding of sudden death in some users, this study highlights an emerging and substantial public health concern.

Currently there is a paucity of primary studies to elucidate the impact on health.

